# Energy Expenditure of Dynamic Submaximal Human Plantarflexion Movements: Model Prediction and Validation by *in-vivo* Magnetic Resonance Spectroscopy

**DOI:** 10.3389/fbioe.2020.00622

**Published:** 2020-06-26

**Authors:** Daniel F. B. Haeufle, Johannes Siegel, Stefan Hochstein, Alexander Gussew, Syn Schmitt, Tobias Siebert, Reinhard Rzanny, Jürgen R. Reichenbach, Norman Stutzig

**Affiliations:** ^1^Multi-level Modeling in Motor Control and Rehabilitation Robotics, Hertie Institute for Clinical Brain Research, University of Tübingen, Tübingen, Germany; ^2^Department of Motion and Exercise Science, Institute of Sport and Movement Science, University of Stuttgart, Stuttgart, Germany; ^3^Motion Science, Institute of Sport Science, Martin-Luther-University Halle, Halle, Germany; ^4^Department of Radiology, University Hospital Halle (Saale), Halle, Germany; ^5^Computational Biophysics and Biorobotics, Institute for Modelling and Simulation of Biomechanical Systems, University of Stuttgart, Stuttgart, Germany; ^6^Stuttgart Center of Simulation Science, University of Stuttgart, Stuttgart, Germany; ^7^Medical Physics Group, Institute of Diagnostic and Interventional Radiology, Jena University Hospital - Friedrich Schiller University, Jena, Germany

**Keywords:** magnetic resonance imaging (MRI), magnetic resonance spectroscopy, ^31^P-MRS, plantar flexion, muscle, biomechanical modeling, energy, validation

## Abstract

To understand the organization and efficiency of biological movement, it is important to evaluate the energy requirements on the level of individual muscles. To this end, predicting energy expenditure with musculoskeletal models in forward-dynamic computer simulations is currently the most promising approach. However, it is challenging to validate muscle models *in-vivo* in humans, because access to the energy expenditure of single muscles is difficult. Previous approaches focused on whole body energy expenditure, e.g., oxygen consumption (VO2), or on thermal measurements of individual muscles by tracking blood flow and heat release (through measurements of the skin temperature). This study proposes to validate models of muscular energy expenditure by using functional phosphorus magnetic resonance spectroscopy (^31^P-MRS). ^31^P-MRS allows to measure phosphocreatine (PCr) concentration which changes in relation to energy expenditure. In the first 25 s of an exercise, PCr breakdown rate reflects ATP hydrolysis, and is therefore a direct measure of muscular enthalpy rate. This method was applied to the gastrocnemius medialis muscle of one healthy subject during repetitive dynamic plantarflexion movements at submaximal contraction, i.e., 20% of the maximum plantarflexion force using a MR compatible ergometer. Furthermore, muscle activity was measured by surface electromyography (EMG). A model (provided as open source) that combines previous models for muscle contraction dynamics and energy expenditure was used to reproduce the experiment in simulation. All parameters (e.g., muscle length and volume, pennation angle) in the model were determined from magnetic resonance imaging or literature (e.g., fiber composition), leaving no free parameters to fit the experimental data. Model prediction and experimental data on the energy supply rates are in good agreement with the validation phase (<25 s) of the dynamic movements. After 25 s, the experimental data differs from the model prediction as the change in PCr does not reflect all metabolic contributions to the energy expenditure anymore and therefore underestimates the energy consumption. This shows that this new approach allows to validate models of muscular energy expenditure in dynamic movements *in vivo*.

## 1. Introduction

Musculoskeletal models are important to predict internal forces and muscular energy expenditure, as it is very challenging to measure both *in vivo* during dynamic movements in humans. Models of muscle contraction dynamics predict forces depending on the length and activity of the muscle tendon unit (MTU) (Winters, 1990; van Soest et al., 1993; Siebert et al., 2003, 2008; Rode et al., 2009; Millard et al., 2013; Haeufle et al., 2014). Models of activation dynamics relate neuronal stimulation to muscle activity (Hatze, 1978; Zajac, 1989; Rockenfeller et al., 2015). Building on these approaches, energy expenditure can be modeled depending on the mechanical and chemical states of the muscles (Umberger et al., 2003; Umberger and Rubenson, 2011; Miller, 2014). However, experimental data to validate such models in humans are rare. Therefore, the aim of this contribution was to perform comprehensive *in vivo* measurements in human muscles including the acquisition of myoelectric activations and mechanical forces during a defined load as well as determining the corresponding chemical energy turnover changes. These latter changes were used to assess energy supply and to compare it with energy expenditure predicted by a dedicated model.

Validating energy expenditure predicted by muscle models is difficult, because muscles not only produce mechanical work—which can be estimated externally from joint kinematics—but also heat. All energy needed for muscle contraction is provided by adenosine triphosphate (ATP) hydrolysis and can be attributed to three dominating processes: (1) the cross-bridge cycling generating mechanical work, (2) pumping calcium ions from the sarcoplasmic reticulum to the myoplasm, and (3) pumping potassium and sodium ions across the sarcolemma (Barclay, 2015). The latter two processes are associated with the transformation of chemical energy into heat and thus reduce the maximum efficiency of a contraction to generate mechanical work (efficiency = mechanical work/energy expenditure) to about 30%. In addition, the maximum efficiency can be further reduced depending on the activation level of the muscle and on the type of contraction (for review see Woledge et al., 1985; Barclay, 2015). To adequately represent these relations in dynamic movement simulations, models of muscular energy expenditure have been developed based on very precise experiments.

Most previous experiments were performed in isolated muscles, especially in frog muscles (Wilkie, 1968; Homsher et al., 1975; Curtin and Woledge, 1979), as these single preparations enable measurements of heat release during contraction. The use of skinned muscle fibers is particularly suitable, as it further enables direct measurement of cross-bridge dependent ATP turnover based on, e.g., a genetically engineered phosphate-binding protein labeled with a coumarin fluorescent probe (Ferenczi et al., 1995) or by monitoring the oxidation of nicotinamide adenine dinucleotide (NADH, which is linked to ADP formation) using spectrophotometric or fluorimetric methods (Takashi, 1979; Barclay, 2015).

Such experiments provide a good foundation for the development of so-called Hill-type models of muscular energy expenditure of individual muscles in terms of stimulation, activity, force, length, and contraction velocity (Umberger et al., 2003; Bhargava et al., 2004; Lichtwark and Wilson, 2005; Umberger and Rubenson, 2011). Integrated into computer simulations, such muscle models allow to estimate the energy consumption of individual muscles during complex dynamic movements, e.g., during human locomotion (Umberger et al., 2003; Umberger and Rubenson, 2011; Miller et al., 2012; Miller, 2014).

The validation of these advanced simulations requires *in vivo* measurements of the muscular energy turnover, for example by using the spirometry technique, which enables quantitation of oxygen consumption (inhaled oxygen volume, VO2). However, although being able to identify the locomotion related energy demand changes (Collins et al., 2015), spirometry fails to assess the energy metabolism in individual muscles (Umberger and Rubenson, 2011). A more suitable approach relies on heat measurements in human muscles (Edwards et al., 1975; González-Alonso et al., 2000). However, their application *in vivo* remains challenging since the following processes have to be taken into account: heat accumulation in muscles, heat removal to the body core mediated by blood circulation and lymph drainage, as well as heat transfer to the skin via convection and conductance (González-Alonso et al., 2000).

On the other hand, dynamic phosphorus magnetic resonance spectroscopy (^31^P-MRS) allows non-invasive, quantitative, localized and simultaneous measurement of different energy metabolites in muscles and thus appears to be a much more favorable technique to obtain appropriate experimental data for the validation of energy turnover models. Besides being able to quantify cell internal phosphate buffer phosphocreatine (PCr) and its intermediate product inorganic phosphate (Pi), ^31^P-MRS also allows measurements of the main energy source adenosine triphosphate (ATP). Acquiring a series of ^31^P-MR spectra during muscle load allows to monitor metabolic processes such as the exercise-induced PCr depletion. During the first seconds of exercise, PCr depletion reflects almost directly the ATP synthesis and immediate ATP demand (Kemp, 2015). Furthermore, the chemical shift of the Pi resonance enables to extract the intra-cellular pH value, because the former is determined by the concentration of free hydrogen ions in the tissue (Heerschap et al., 1999; Rzanny et al., 2016). Therefore, inspecting the load evoked pH changes allows differentiation between alternative ATP synthesis pathways such as alkaline creatine kinase reaction (pH increase) and anaerobic glycolysis (pH decrease). When the pH-increase starts to slow down, the ATP synthesis via glycolysis becomes more dominant. Thus, from the point when the pH reaches its maximum, the ATP demand cannot be estimated anymore based on PCr depletion.

Against this background, the aim of this study was to quantify the ATP turnover rate in the human calf muscle based on PCr depletion of dynamic exercise and use these data to validate a Hill-type model of muscular energy consumption. We hypothesized that the energy expenditure derived from the ^31^P-MRS data matches the energy expenditure predicted by the Hill-type model in a repetitive submaximal exercise for approximately 25 s and will drifting apart when the pH value reaches its maximum. Therefore, we focus on these first 25 s as our validation phase. A model validated by this approach can be used in future studies to predict energy expenditure for sub-maximal dynamic contractions.

## 2. Experiments

All measurements were conducted in one healthy male subject (age: 36 y., height: 1.94 m, weight: 92 kg, BMI: 25) on the same day. The subject was informed about the aims and risks of the study and gave his written consent before the experiments started. The study was approved by the local ethics committee of the University Hospital of Jena, Germany.

**Figure 1:**
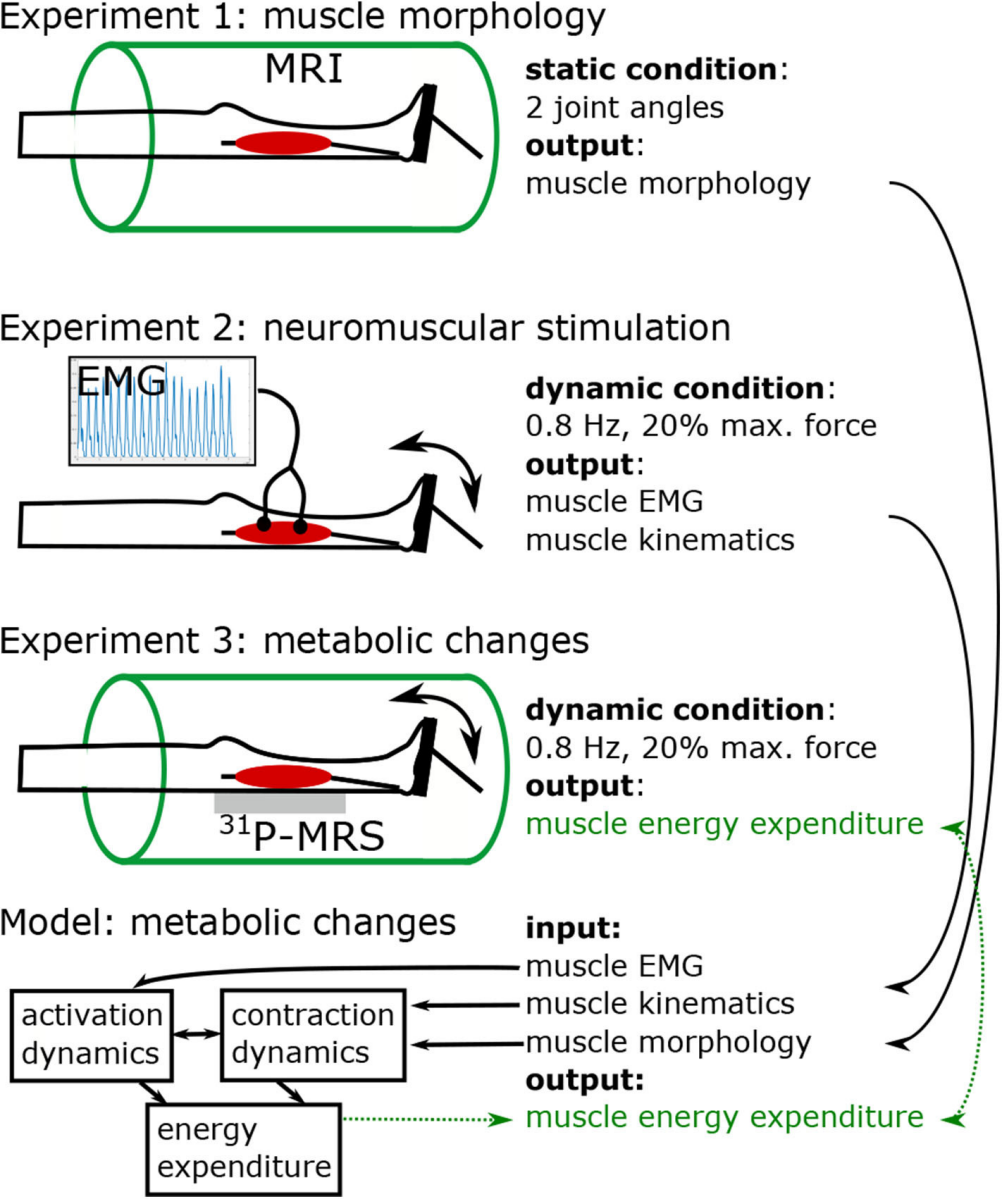
Study design, including three experiments and model based simulation of energy turnover changes in loaded calf muscle. Experiment 1: Structural MRI to determine muscle volume, muscle-tendon-unit length, fiber length, fiber angle, and lever arm in two joint angles (neutral, and extended). Morphological data was used to individualize the leg-foot geometry in the muscle model. Experiments 2 (EMG) and 3 (^31^P-MRS) were performed in a contracting calf muscle at 20% of the *single repetition maximum* force with 0.8 *Hz* pedaling cadence. Data from Experiment 2 were used to determine dynamic muscle length (based on recorded joint angle) and myoelectrical stimulations (EMG) during the exercise. The ^31^P-MRS data were used to quantify the ATP turnover and to determine the muscular energy expenditure, which was compared with the output of model based simulations. The model receives input from experiment 1 in terms of muscle parameters and experiment 2 in terms of dynamic muscle length and EMG.

Figure 1 shows the study protocol consisting of three separate experiments. In experiment 1, high resolution MRI scans were performed to obtain the anatomy of the right foot and calf (e.g., muscle volume, internal muscle moment arm). Experiments 2 and 3 were conducted to acquire electromyography (EMG) and ^31^P-MRS data in the M. gastrocnemius medialis (GM) during a defined repetitive plantarflexion exercise. While using the same load regimes, experiments 2 and 3 were performed separately in order to avoid interferences between the EMG and ^31^P-MRS acquisitions.

All measurements were performed in supine position with an in-house, MR-compatible pedal ergometer, which is equipped with appropriate sensors and a graphical user interface to monitor the angulation and force magnitudes (Tschiesche et al., 2014; Stutzig et al., 2017). The foot was angulated between 0° (foot sole perpendicular to the shank) and 25° (plantarflexion) and the leg was fixed using a belt attached approximately 3 cm proximal to the patella. Prior to the main exercises, the volunteer performed a standardized warm-up consisting of 10 submaximal isometric plantarflexions. Next, the subject performed one isometric (no joint movement) *maximum voluntary contraction* at rest angle (later used to normalize EMG) followed by three *single repetition maximum* contractions (with joint movement) separated by 1 min each (used for determining the submaximal force level for the main experiment).

MR measurements were conducted on a clinical 3 T whole-body MR scanner (Magnetom PRISMA fit; bore size, 60 cm; VE11B, Siemens Healthcare, Erlangen, Germany). Scans were performed using either a flexible phased-array surface coil (Siemens Healthcare, Erlangen, Germany) or a double-tuned (^1^H/^31^P) transmit/receive flexible surface coil with an integrated ^31^P-loop (diameter 11 *cm*, RAPID BioMedical; Rimpar, Germany). The coils were wrapped around the right lower leg.

### 2.1. Experiment 1 - Determination of Muscle Morphology

To determine geometrical parameters of the M. gastrocnemius medialis, which are needed for the muscle model (see section 3), we collected two high resolution T_1_ weighted MRI data sets in two positions (ϕ = 0° neutral ankle position and ϕ = 25° ankle flexion). Each dataset was generated by assembling three, separately acquired and overlapping MRI data volumes covering, first, the upper part of lower leg with the knee joint, second, the middle lower leg part and, third, the lower part of lower leg with the ankle joint and feet. We used a 3D VIBE sequence with an isotropic spatial resolution of 0.8 *mm*^3^ (TR/TE = 4.65/2.46 *ms*, flip angle: 8°, 3 averages, matrix size: 320 × 320, FOV_*AP* × *FH*_: (256 × 256) *mm*, 160 sagittal slices, 0.8 *mm* slice thickness, TA: 4 *min*). The assembling of data sets was performed by using an automatic registration routine available on the analysis console of the MR scanner. The assembled 3D data set was saved as a DICOM image series and manually segmented by using OsiriX MD software (Pixmeo SARL, Bernex, Switzerland). Based on this analysis, muscle volume (*V*), muscle-tendon-unit length (*l*_*MTU*_), muscle fiber length (*l*_*CE, opt*_), pennation angle (α), and lever arm (*r*) were determined for the M. gastrocnemius medialis (see also Table 1 and section 3.1).

**Table 1:** Subject specific model parameters.

**Symbol**	**Meaning**	**Value for model of *M. gastrocnemius***	**Source**
*F*_max_	Maximum isometric muscle force	1474 *N*	MRI (Equation 12)
*l*_CE, opt_	Optimal fiber length	0.0574 *m*	MRI (Equation 10)
*r*_fib_	Ratio of fast to slow twitch fibers	0.45	Saltin and Gollnick, 1983
*A*_rel, 0_	Hill-constant for force-velocity relation	0.28	Umberger et al., 2003
*B*_rel, 0_	Hill-constant for force-velocity relation	3.36	Umberger et al., 2003
*M*	Muscle mass	0.3587 *kg*	MRI (Equation 13)
*l*_SEE, 0_	Slack length of the tendon	0.2573 *m*	MRI (Equation 11)
*V*	Muscle volume	379.89·10^−6^*m*^3^	MRI

*All other parameters are generic parameters taken from literature as listed in the Electronic Supplementary Material*.

**Figure 2:**
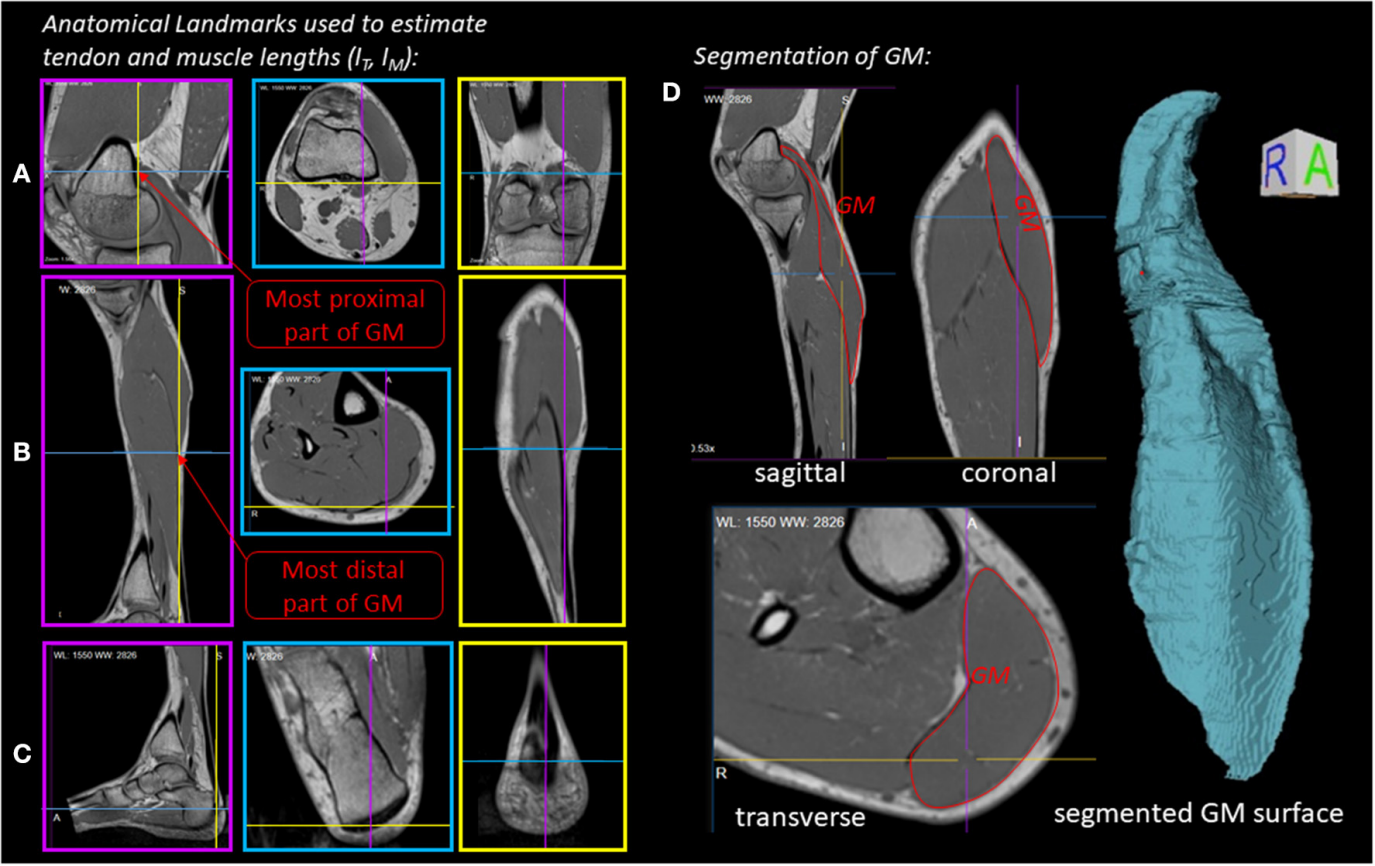
Experiment 1—Determination of muscle morphology: **(A–C)** Sagittal (magenta boxes), transverse (blue boxes) and coronal (yellow boxes) MRI planes illustrating anatomical landmarks (indicated by line cross-sections), which were identified to determine the tendon (*l*_*SEE*_) and muscle lengths (*l*_CE_). **(D)** Manually segmented GM muscle and corresponding GM surface.

Figure 2 illustrates the anatomical landmarks (Figures 2A–C), which were identified on MR images to determine the tendon *l*_*SEE*_ and muscle fiber lengths *l*_*CE*_, as well as a GM surface (Figure 2D), which was manually segmented and used to calculate the muscle volume *V*. The tendon length *l*_*SEE*_ was determined as a distance between the insertion of the Achilles tendon (Figure 2C) to the most distal GM part landmark (Figure 2B), which was accessed by identifying the first transverse MRI slice containing the GM muscle. The muscle length *l*_*MTU*_ was estimated as a distance between the most distal GM part landmark (Figure 2B) and the landmark indicating the GM origin at the medial condyle of the femur (Figure 2A). The pennation angle α = 27° and the fascicle length *l*_CE, opt_ = 57 *mm* were determined based on sagittal plane, solely. Due to the fact that the structures were hard to find in the MR images, we compared our results with studies that examined the architecture of the gastrocnemius medialis using ultrasound (Narici et al., 1996; Arampatzis et al., 2006). The acquired parameters show a good agreement with data reported in literature especially for fiber length. The pennation angle is slightly higher than the data found in the literature. An architectural deviation might be due to compression by the flexible coil which was positioned around the shank in our study.

### 2.2. Experiment 2 - Determination of Neuromuscular Stimulation

The dynamic exercises in experiment 2 consisted of dynamic plantarflexion series (3 min exercise + 27 min recovery) at 20% of the mean force level achieved in the three *single repetition maxmimum* trials. The subject was asked to maintain a pedaling frequency of 0.8 Hz while being assisted by a metronome. In experiment 2, surface EMG and joint angle were recorded to be used as input to the muscle model (Figure 1).

Surface EMG data were recorded using a previously described electrode positioning scheme (Stutzig and Siebert, 2016) and by following the recommendations of Barbero et al. (2012). The skin under the electrodes was shaved, abraded and cleaned with an alcohol solution (according to SENIAM recommendations, Hermens et al., 2000). Two bipolar Ag/AgCl electrodes were affixed with an inter-electrode distance of 20 *mm*. The data were recorded with a sample rate of 2 *kHz*, pre-amplified (factor 1000), bandpass filtered (10–500Hz), and rectified by a Biopac MP160 system (Biopac Systems, Goleta, California, USA). The EMG data were normalized to the EMG maximum obtained during the *maximum voluntary contraction* trial.

### 2.3. Experiment 3 - Determination of Metabolic Changes

We employed dynamic phosphorus magnetic resonance spectroscopy (^31^P-MRS) to measure the change in phosphocreatine (PCr) concentration ([PCr], the brackets indicate the concentration of PCr), reflecting the ATP concentration change in the muscle. The movements were identical as in experiment 2.

**Figure 3:**
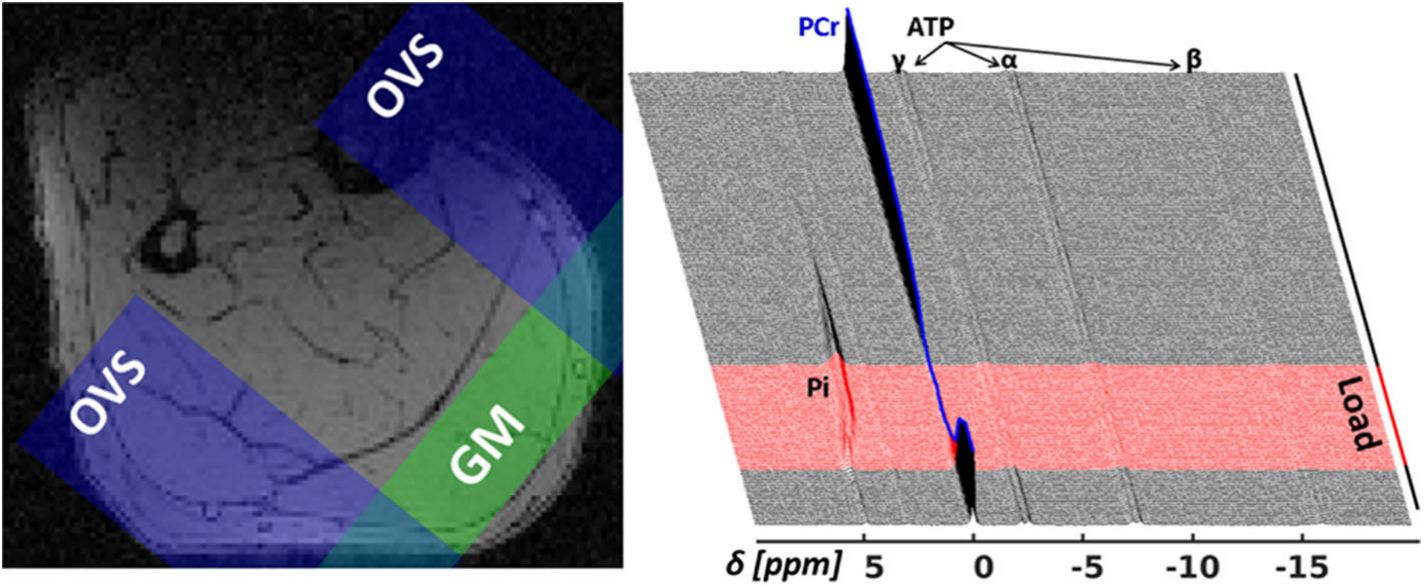
**Left**: Transverse T_1_-weighted gradient-echo image (3D T_1_-weighted gradient-echo MRI, TR/TE = 10/6.15 ms) with selected ^31^P-MRS slice in M. gastrocnemius medialis (GM, green area) and outer volume suppression bands (OVS, blue areas) selected to suppress signal contributions from adjacent tissue. **Right**: “Stack plot” of dynamic ^31^P-MR spectra series from GM muscle. The spectra acquired during the 3 *min* load phase (red graphs in the right chart) show rapid PCr decrease during the validation phase as well as a subsequent, significant resonance frequency shift of inorganic phosphate (Pi) due to a strong, anaerobic glycolysis related pH decrease. Please note that this paper focuses on the first steep decrease of PCr in the validation phase (*t* < 25 s).

An in-house developed ^31^P-MRS pulse sequence, dubbed MUSCLE (MUlti SliCe Localized Excitation (Moll et al., 2018); TR = 5, 000 *ms*; flip angle: 50°; acquisition delay between excitation RF pulse and acquisition: 1.3 *ms*; excitation pulse duration/bandwidth: 1, 600 μs/2.5 *kHz*), was used to acquire series of 360 single ^31^P-MR spectra in a 16 *mm* thick slice covering the M. gastrocnemius medialis (see Figure 3 for slice positions). In an entire ^31^P-MR spectra series (see the right chart of Figure 3), 24 spectra were measured during rest (2 *min*), followed by the acquisition of 36 spectra during load (3 *min*), and 300 spectra during recovery (25 *min*). The series was acquired with a temporal resolution of 5 s. Prior to acquiring the dynamic ^31^P-MRS data, a fully recovered resting state spectrum (reference spectrum) was collected with TR of 15 s.

All MR spectra were corrected for zero- and first-order phase errors, frequency shifts as well as baseline distortions by using an in-house MATLAB script and were quantified with an AMARES routine with prior knowledge for linewidth, frequency and peak shape (Vanhamme et al., 1997) included in the jMRUI 5.2 software (Stefan et al., 2009). Whereas the PCr peak at 0 *ppm* was fitted as a single Lorentzian line, the α-ATP (at −7.52 *ppm*) and γ-ATP (−2.48 *ppm*) doublets and the β-ATP triplet (−16.26 *ppm*) were quantified with pre-constrained linear combinations of two and three Lorentzian lines, respectively. Absolute PCr concentrations ([PCr]) were determined from the reference spectrum by normalizing the peak intensities (I_PCr_) with the γ-ATP intensity (I_γ-ATP_) and by assuming a resting state muscle ATP concentration of 8.2 *mmol*/*l* ([*PCr*] = 8.2 *mmol*/*l* × I_PCr_/I_γ-ATP_) (Kemp et al., 2007).

### 2.4. Analysis of Energy Expenditure in the Validation Phase

The energy demand to maintain muscle performance is met by the breakdown of ATP, which is transferred to the myosin head of the muscle fibers during the cross-bridge cycle with subsequent hydrolysis (Equation 1; Barclay and Weber, 2004). Since ATP depletion is quickly compensated via the creatine kinase reaction (CK; Equation 2), there are usually no ^31^P-MR spectroscopically detectable ATP changes that could be used to assess the energy demand. However, in the validation phase (*t* < 25 s), the ATP synthesis rate can be derived from the depletion rate of PCr, which is consumed at the same rate as the rate of the ATP synthesis:

(1) $$\begin{array}{l} ATP^{4-}+H_{2}O⇌ADP^{3-}+HPO_{4}^{2-}+H^{+} \end{array}$$

(2) $$\begin{array}{l} PCr^{2-}+ADP^{3-}+H^{+}⇌ATP^{4-}+Cr \end{array}$$

Therefore, the energy expenditure *dE*/*dt* can be determined from the measured PCr breakdown rate *d*[*PCr*]/*dt* and the enthalpy of ATP hydrolysis △_*R*_*H*(*ATP*)

(3) $$\begin{array}{l} \frac{dE}{dt}=\frac{d\left[PCr\right]}{dt}\cdot {△}_{R}H\left(ATP\right) \end{array}$$

with ${△}_{R}H\left(ATP\right)=21kJmol^{-1}$ (Kodama, 1985; see Table 4, for temperature of 38°C). This indicates that an ATP hydrolysis rate of 1 *mmol l*^−1^s^−1^ is associated with a release of 21*J* reaction heat per liter cell water *per second*. To calculate the energy released per kilogram of muscle wet weight, we used a conversion factor of $c_{\text{vol}\to \text{mass}}=0.68lkg^{-1}$ provided by Kemp et al. (2007, Table 1, second row):

(4) $$\begin{array}{l} 0.68\text{ lk}{\text{g}}^{-1}\cdot 21\text{ J}{\text{l}}^{-1}{\text{s}}^{-1}=14.28\text{ Jk}{\text{g}}^{-1}{\text{s}}^{-1} \end{array}$$

By approximating the rate of change of PCr concentration by

(5) $$\begin{array}{l} \frac{d[PCr]}{dt}\approx \frac{{\left[PCr\right]}_{i+1}-{\left[PCr\right]}_{i}}{t_{i+1}-t_{i}} \end{array}$$

the energy release can directly be calculated from the ^31^P-MRS data (with Equation 4)

(6) $$\begin{array}{l} \frac{dE}{dt}=\frac{\frac{d\left[PCr\right]}{dt}}{1\;\text{mmol }l^{-1}s^{-1}}\cdot 14.28\text{ Jk}{\text{g}}^{-1}{\text{s}}^{-1}. \end{array}$$

Please note that this relation only holds in the validation phase (*t* < 25 s) where the ATP synthesis is dominated by the depletion of PCr.

## 3. Model

To predict muscular energy expenditure, we combined the following three existing models describing: (a) the contraction dynamics based on a four-element Hill-type muscle model including series elastic damping characteristics (Haeufle et al., 2014), (b) the activation dynamics (Umberger et al., 2003), and (c) the energy consumption (Umberger et al., 2003).

The **MTU contraction dynamics** is based on a Hill-type muscle model (Haeufle et al., 2014), which considers force-length-velocity characteristics of the fibers (contractile element CE), parallel connective tissue elasticities (parallel elastic element PEE), tendon elasticity (serial elastic element SEE), and tendon damping characteristics (serial damping element SDE). The model has one internal degree of freedom represented by the length of the contractile element *l*_CE_. Thus, the contraction dynamics of the MTU is described by a differential equation

(7) $$\begin{array}{l} {\overset{\cdot }{l}}_{\text{CE}}=f_{\text{CE}}\left(l_{\text{CE}},l_{\text{MTU}},{\overset{\cdot }{l}}_{\text{MTU}},a\right), \end{array}$$

where the fiber contraction velocity (${\overset{\cdot }{l}}_{\text{CE}}$) is a function of the fiber length (*l*_CE_), the length (*l*_MTU_), and contraction velocity of the muscle tendon unit (${\overset{\cdot }{l}}_{\text{MTU}}$) and the muscle fiber activity (*a*). The model predicts the muscle fiber force *F*_CE_ and is described in detail in Haeufle et al. (2014).

The **activation dynamics** model considers the complex biochemical relation between neuronal stimulation of the muscle, *u*, and muscle activity *a*, where 0.001 ≤ *a* ≤ 1 and 0 ≤ *u* ≤ 1 are normalized variables. These processes can be approximated by a first order differential equation (He et al., 1991; Umberger et al., 2003)

(8) $$\begin{array}{l} ȧ=\left(u-a\right)\left(\left(\frac{1}{\tau_{\text{ACT}}}-\frac{1}{\tau_{\text{DEACT}}}\right)u+\frac{1}{\tau_{\text{DEACT}}}\right), \end{array}$$

with different time constants considering faster activation τ_ACT_ = 58.8 *ms* than deactivation τ_DEACT_ = 64.8 *ms* of muscle fibers (calculated by Equation 4 in Umberger et al., 2003 with *%FT* = *r*_fib_ = 0.45 in accordance to Saltin and Gollnick 1983).

The model for the **energy expenditure** of the MTU considers the non-linear relation between muscle activation, contraction state, and energy expenditure, as well as the fiber type composition (Umberger et al., 2003). It calculates the total energy rate

(9) $$\begin{array}{l} \dot{E}={\dot{h}}_{\text{AM}}+{\dot{h}}_{\text{SL}}+{\dot{w}}_{\text{CE}}, \end{array}$$

as the sum of three components: the activation heat rate and maintenance heat rate ḣ_AM_ = *f*_hAM_(*l*_CE_, *u, a*), the shortening-lengthening heat rate ${ḣ}_{\text{SL}}=f_{\text{hSL}}\left({\overset{\cdot }{l}}_{\text{CE}},l_{\text{CE}},u,a\right)$, and the mechanical work rate ${ẇ}_{\text{CE}}=f_{\text{wCE}}\left({\overset{\cdot }{l}}_{\text{CE}},F_{\text{CE}}\right)$. All state variables required to calculate the energy expenditure are provided by the model of the contraction and activation dynamics of the MTU.

All model equations were implemented in Matlab Simulink R2018a (Mathworks Inc.). The model is provided open-source via github (https://github.com/daniel-haeufle/macroscopic-muscle-model) and attached as Electronic Supplementary Material.

### 3.1. Model Parameters

Most of the parameters of all three model parts were taken from the original sources [contraction dynamics (Haeufle et al., 2014), activation dynamics (He et al., 1991; Umberger et al., 2003), and energy expenditure (Umberger et al., 2003)]. The muscle specific parameters are listed in Table 1 and the complete parameter set is provided with the model in the Electronic Supplementary Material (Haeufle, 2020).

We set the optimal length of the contractile element equal to the fiber length at the pedal rest position

(10) $$\begin{array}{l} l_{\text{CE,opt}}=l_{\text{CE}}\left(\phi =0^{\circ }\right)=0.057\text{ m}. \end{array}$$

The slack length of the serial elastic element was calculated as

(11) $$\begin{array}{l} l_{\text{SEE,}0}=l_{\text{MTU}}\left(\phi =0^{\circ }\right)-l_{\text{CE,opt}}=0.315\text{ m}-l_{\text{CE,opt}}=0.257\text{ m}. \end{array}$$

The maximum isometric force *F*_*max*_ was determined based on the muscle volume (*V*) (Holzbaur et al., 2005) as

(12) $$\begin{array}{l} F_{\text{max}}=\sigma \frac{V}{l_{\text{CE.opt}}}\text{cos}\left(\alpha \right)=1474\text{ N}, \end{array}$$

with the overall maximum muscle stress σ = 0.25 *MPa* (Umberger et al., 2003) and pennation angle α = 27°, which was obtained from anatomic MRI data. The mass of the muscle was calculated to be

(13) $$\begin{array}{l} M=V\rho =0.359\text{ kg}, \end{array}$$

with the muscle tissue density ρ = 1059.7 *kg*/*m*^3^ (Méndez et al., 1960). We set the fiber type composition (ratio of fast to slow twitch fibers) to *r*_fib_ = 0.45, which corresponds to trained distance runners with the mean age of 35 years (Saltin and Gollnick, 1983) matching our subject. This results in Hill parameters according to Umberger et al. (2003) of *A*_rel, 0_ = 0.1+0.4*r*_fib_ = 0.28 and *B*_rel, 0_ = 12*A*_rel, 0_ = 3.36.

As the parameters determined from the MRI images are prone to measurement errors, we estimated the effect of variations in these parameters on the calculation of the total energy by means of a Monte Carlo simulation (100 runs). For this, we estimated errors of Δ*l*_CE, opt_/*l*_CE, opt_ = 10%, $\Delta l_{\text{MTU}}\left(\phi =0^{\circ }\right)/l_{\text{MTU}}\left(\phi =0^{\circ }\right)=5\%$, and Δα/α = 10%. The mean effect of these variations is indicated by the light-blue area around the simulation results in **Figure 5** and appears to be marginal (<1.3%) with respect to the magnitude of the simulated energy expenditure.

### 3.2. Simulation

The simulation reproduced the experimental conditions of the dynamic plantarflexion movements (experiments 2 and 3). We derived the MTU kinematics *l*_MTU_(*t*) and ${\overset{\cdot }{l}}_{\text{MTU}}\left(t\right)$ from the foot pedal angle ϕ(*t*) as recorded by the ergometer. We assumed a constant muscle lever arm of *r* = 0.064 *m* as determined from the MRI data set (experiment 1). We fitted a tenth-order polynomial to the angle data which allowed to analytically calculate a smooth derivative for ${\overset{\cdot }{l}}_{\text{MTU}}\left(t\right)$. For the muscle stimulation signal *u*(*t*), we used the rectified and normalized EMG signal measured in experiment 2.

The boundary conditions for the simulation were chosen in accordance with the experimental data. From the initial joint angle ϕ(*t* = 0) = 3.13° we calculated the initial MTU length as *l*_MTU, init_ = 0.311 *m*. The initial muscle activity was *a*_init_ = 0.005, corresponding to the initial stimulation *u*(*t* = 0) = 0.005 from the normalized EMG signal. This allows to determine the boundary condition for the muscle model's internal degree of freedom, the muscle fiber length *l*_CE, init_ = 0.052 *m*: by assuming static force equilibrium at *t* = 0, all velocity dependent terms in the muscle model vanish and the length of the contractile element can be calculated from the force-length curve of the CE and SEE with the parameters *l*_MTU, init_ and *a*_init_ (see Haeufle et al., 2014 for more details).

The differential equations were solved with ODE23s Simulink built-in variable time step solver with relative tolerance of 1·10^−4^.

## 4. Results

**Figure 4:**
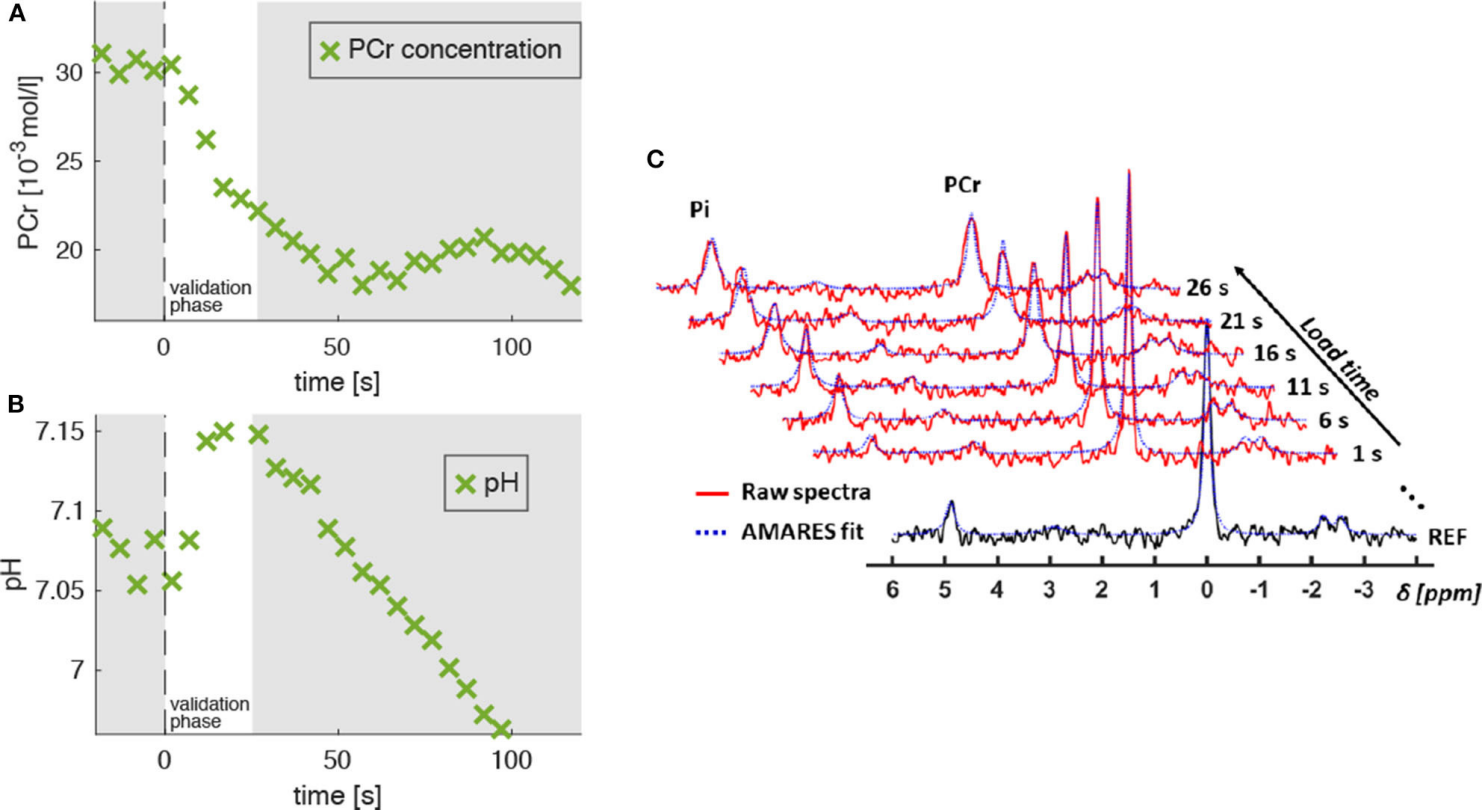
Evolution of PCr concentration ([PCr], **A**) and pH level **(B)** extracted from ^31^P-MR spectra, which were acquired in the M. gastrocnemius medialis during dynamic plantarflexion exercise (experiment 3, section 2.3). The time point *t* = 0 s marks the onset of the exercise. Highlighted is the validation phase (*t* = 0…25 s) for which we hypothesized agreement between experiment and model. During this phase, the PCr concentration reveals a continuous decrease indicating ATP re-synthesis and, thus, energy consumption in the muscle (see section 2.4). During the validation phase the energy supply is dominated by the PCr depletion. The pH decrease for *t* > 25 s reflects the H+ accumulation during the anaerobic glycolysis. Subplot **(C)** shows the raw spectra (red graphs) and corresponding AMARES fits (blue dashed graphs), obtained during the validation phase. The black graph in the sublot **(C)** shows a spectrum obtained prior to exercise.

Figure 4A shows a typical PCr evolution for an intense muscle exercise. It starts with a strong PCr depletion during the validation phase (*t* < 25 s), which levels off approximately after 60 s. The H^+^ consumption accompanying the initial PCr breakdown is indicated by a continuous pH increase from 7.05 to 7.1 (Figure 4B) followed by a strong pH decrease (H^+^ accumulation) in the later exercise phase, which is dominated by anaerobic glycolysis.

**Figure 5:**
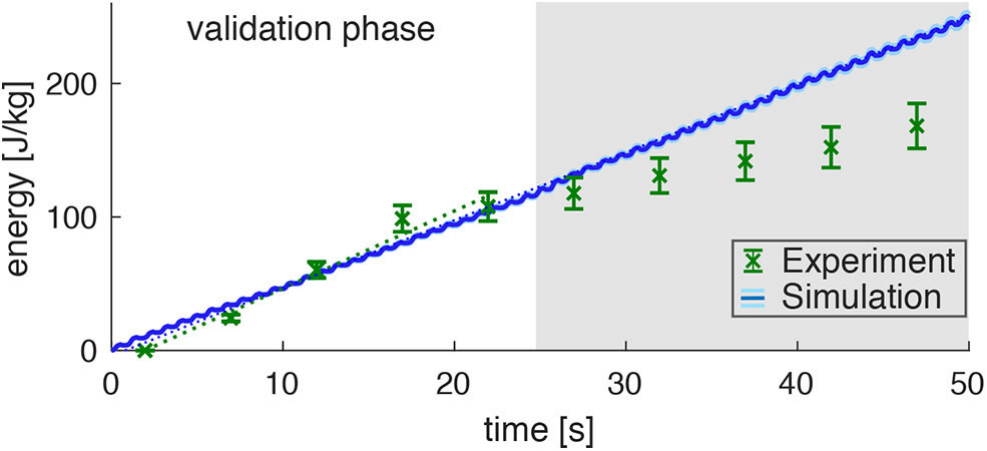
Prediction of the **energy consumption by the model** (mean: blue line, standard deviation: very small light-blue shade around the blue line) compared to the measured energy consumption in the experiments (derived from the PCr decay; green data points) for dynamic plantarflexion. The model predicts the energy rate (${Ė}_{\text{sim}}=5.081\pm 0.064Jkg^{-1}s^{-1}$, linear fit, dotted blue line) as the sum of activation-maintenance heat, shortening-lengthening heat, and mechanical work performed by the muscle fibers: Ė = ḣ_*AM*_ + ḣ_*SL*_ +ẇ_*CE*_. The **experimental data** shows a similar energy rate (${Ė}_{\text{exp}}=5.8\pm 1.8Jkg^{-1}s^{-1}$, linear fit, dotted green line) during the first 25s (validation phase). During this time, the PCr decay dominates the ATP re-synthesis. Afterwards, the aerobic and anaerobic metabolism additionally contributes (see the progressing pH depletion in Figure 4B). Hence, after 25s not the whole energy consumption is reflected by the measured [PCr] reduction. The error margin for the simulation (light blue) was determined by a Monte-Carlo simulation based on the estimated errors of the subject specific parameters determined in Experiment 1. The error for the experimental energy consumption estimates the maximum error of the ^31^P-MRS.

Assuming equivalent rates of PCr consumption and ATP synthesis during the validation phase (*t* < 25s) of the exercise, the amount of energy provided via the ATP hydrolysis can be calculated according to Equation (6). This reveals a steep accumulation of energy with a rate of ${Ė}_{\text{exp}}=5.8\pm 1.8Jkg^{-1}s^{-1}$ (Figure 5, linear fit to the experimental data points with *t* < 25 s, confidence interval 95%). During the later exercise phase, the energy supply is dominated by anaerobic glycolysis and thus cannot be estimated from PCr evolution.

The energy expenditure rate predicted by the muscle model amounted to ${Ė}_{\text{sim}}=5.081\pm 0.064Jkg^{-1}s^{-1}$ (see the blue graph in Figure 4, linear fit). This is in good agreement to the experimental data for the validation phase of the exercise (*t* < 25 s). The mismatch after the validation phase was expected, as here PCr consumption and ATP synthesis do not have equivalent rates as energy supply is expected to be dominated by anaerobic glycolysisdue. This is indicated by a progressing deviation between simulation and experiment, as they were obtained from the model and PCr time courses, respectively.

Please note, that the time resolution of the simulation is much higher than the time resolution of the experimental data and therefore predicts the small subtle variations in energy rate within every plantarflexion cycle, which is not accessible by the experiment. The estimated model prediction error, as determined by parameter variation, is $\frac{\Delta E}{E}<0.013$ (not even visible in Figure 5 during the validation phase).

## 5. Discussion

We presented a model that predicts the muscular energy expenditure during dynamic exercise of the human calf muscle and validated this model with metabolic adaptations obtained from *in vivo* ^31^P-MRS experiments. Generic parameters were taken from literature and subject specific parameters—which reflect the anatomy of the examined subject—were extracted from MRI scans, leaving no free parameter which would require fitting. In order to reproduce the exercise conditions in model based simulations, the model input variables, i.e., the muscle-tendon-unit kinematics and the neuronal muscle stimulation, were defined based on the real experimental data, i.e., on the pedal angulation and EMG recordings. For model validation, we used the spectroscopically extracted rate of PCr depletion, which reflects the ATP synthesis rate in the validation phase of the exercise and, thus, the energy consumption in the muscle.

The main question of the present study whether the applied model can properly reflect the energy expenditure in the loaded muscle can be positively answered in light of the matching between the predicted and experimentally determined energy supply in the validation phase of the exercise.

The increasing mismatch between model prediction and experimental data for *t* > 25 s was expected, as the PCr break down reflects ATP hydrolysis only during the validation phase of the exercise (Heerschap et al., 1999). The PCr depletion is associated with proton consumption, i.e., the cytosolic pH increase. This situation starts to change in the later phase, where the ATP synthesis is dominated by the anaerobic glycolysis and oxidative phosphorylation. While the latter pathway cannot be directly assessed by means of ^31^P-MR spectroscopy, the onset of the former process is reflected in a steep pH decrease starting approximately 25 s after the exercise begin (see Figure 4B). Our observations are in line with a previous study of Rossiter et al. (2002), who also reported a pH increase from 7.06 to 7.11 after 30 s of exercise onset (see also review by Kemp, 2015). Therefore, the turning point of the pH evolution can be used to set the limits for the time period, during which the predicted and experimentally determined energy supply can be compared. However, as indicated by the constant β-ATP intensities in the ^31^P-MR spectra series, the net cytosolic ATP concentration remained constant throughout the entire exercise period. Thus, we conclude a steady state of ATP hydrolysis and ATP production during the entire time course of the experiment. Furthermore, we assume, as long as the cytosolic pH increases that the ATP production results almost solely from the break-down of PCr.

However, this time window of *t* < 25 s likely depends on the exercise intensity. At higher intensities (e.g., >20% of *single repetition maximum*), the PCr breakdown rate will increase and the glycolytic pathways start earlier (<10 s at >90% of *single repetition maximum*, Barclay 2015).

The advantage of our approach is the possibility to validate the model prediction at the level of individual muscles. Previously, energy expenditure could either be measured *in vivo* on the organism level (Waters and Mulroy, 1999; Bassett and Howley, 2000; Levine, 2005; Corbett et al., 2008; Au et al., 2009) or estimated from temperature changes for muscle groups (Edwards et al., 1975; González-Alonso et al., 2000; Reitman, 2018). Attempts have been made to combine these measures with musculoskeletal models to estimate how the total energy consumption is distributed between muscles (Umberger, 2010; Miller et al., 2012; Miller, 2014; Dorn et al., 2015; Koelewijn et al., 2019). However, for this purpose it was necessary to estimate and consider the energy consumption outside of muscles as well as the load sharing between muscles. Measuring energy consumption in individual muscles was only possible in *in vitro* experiments by using isolated animal muscles (Homsher et al., 1975; Curtin and Woledge, 1979). Only one other method has been proposed to measure individual muscular energy consumption in animals *in vivo* (Marsh, 2006). This method estimates energy consumption from marker particles injected into the blood which accumulate in muscular tissue over time—with the limitation that the measurement can only be performed by dissecting the animal and, thus, allows to estimate an accumulative effect only for the active movement phase shortly before life termination.

### 5.1. Limitations

#### 5.1.1. Experimental Limitations

Due to the strong magnetic field of the MR scanner, EMG measurements had to be performed in separate trials outside of the scanner. Hence, only very standardized repetitive movements can be investigated and used to validate the model. In addition, the relatively low temporal resolution of ^31^P-MRS series (5 s) prevents the validation of the fine-grained temporal prediction of the muscle model, which resolves the energetic difference between flexion and extension in each cycle (Figure 5). Furthermore, applying the ^31^P-MRS method to a specific muscle requires careful consideration of partial volume effects from other muscles. In this study, the relatively large thickness of the excited spectroscopic slice as well as the leg movements might provoke contaminations of metabolic adaptations in the M. gastrocnemius medialis by contributions from the adjacent muscles M. gastrocnemius lateralis or M. soleus. However, these effects appear to be marginal as the spectroscopic slice captures almost exclusively the M. gastrocnemius medialis (Figure 3). In our study, we used a ^31^P-MRS sequence equipped with a localization scheme, which follows the DRESS technique (Depth-REsolved Surface coil MRS) of Valkovič et al. (2014). A more accurate localization of muscle of interest can be achieved by using a localized semi-LASER dynamic ^31^P-MRS method with selection of well-defined spectroscopic volumes (Fiedler et al., 2015; Niess et al., 2018). However, we decided against the latter approach, since it relies on a spin echo based localization scheme and thus is more susceptible to signal attenuations due to accelerated transverse relaxation of phosphorous metabolites (Meyerspeer et al., 2003).

#### 5.1.2. Robustness Concerning Experimental and Physiological Variations

The model validation is based on the experimental data set of a single subject and one sub-maximal stimulation level only. The model reproduces the experimental data very well. However, subjects with other muscle fiber compositions, training states, etc. could show a different energy expenditure [up to ca. 10% standard deviation for level walking between subjects (Koelewijn et al., 2019)]. Hence, the robustness of our findings concerning inter-individual differences in physiology and anatomy, and for different stimulation levels has to be evaluated in future studies.

#### 5.1.3. Force-Level Validation

The study is currently missing simultaneous validation of the force produced by the muscle. This would require either a direct measurement of the M. gastrocnemius force—which is ethically and technically difficult—or a complete modeling, ^31^P-MR spectroscopic and EMG assessment of all lower leg muscles and subsequent comparison of simulation results with the force time course, which can be measured in the pedal ergometer. For this, also a validation of the muscle's maximum force, e.g., via a dynamometer would be required (Menegaldo and Oliveira, 2012). Therefore, we currently have to rely on the literature, where the muscle model has been validated with respect to force generation in experiments with isolated animal muscles (Günther et al., 2007; Mörl et al., 2012).

#### 5.1.4. Activation Dynamics

Furthermore, the model currently includes activation dynamics, which do not consider fiber-length dependencies. There was a controversy over which activation dynamics are physiologically more relevant. The length-dependent *Hatze* activation dynamics is strongly supported by experiments on isolated muscles (Rockenfeller et al., 2015). However, if EMG data is used as input to the muscle model, the simpler *Zajac* activation dynamics may be more appropriate (Kramer, 2012; Menegaldo and Oliveira, 2012). We used the latter in analogy to the original publication of the energy model (Umberger et al., 2003).

#### 5.1.5. Model Parameters

With respect to the model parameters, we confirmed that a variation of the experimentally determined muscle parameters—according to their respective error—has a small effect on the model's energy prediction ($\frac{\Delta E}{E}<0.013$, Figure 5, light-blue shaded area around the simulation results). The normalization of the EMG signal may further influence the predicted energy. Here, we used the *maximum voluntary contraction*, a standard method for normalization (Menegaldo and Oliveira, 2012). The parameters used to link enthalpy rate with ATP hydrolysis were taken from *in vitro* experiments under defined conditions (Kodama, 1985) which may differ in *in vivo* contractions. Moreover, maintenance and shortening heat rates and the fiber type composition largely influence energy output rate. Such parameters are based only on a very small number of studies, and they are variable because different muscle preparations, contraction protocols and methods of measuring were used. Bolstad and Ersland (1978) investigated the rate of temperature rise during voluntary isometric contractions in human M. soleus, M. sarcospinalis, and M. biceps brachii. They found that the heat rate of fast twitch fibers is approximately six times that of slow twitch fibers. Moreover, heat rates depend on temperature (Barclay, 2015) which varies greatly between studies. Even for similar muscle preparations (e.g., mouse M. soleus) at given temperature (20°) the rate of ATP turnover varies by about 80% in different studies (Crow and Kushmerick, 1982; Barclay et al., 2010). Furthermore, muscle activation impacts muscle efficiency and activation costs (Lewis and Barclay, 2014; Barclay, 2015). This variability makes it difficult to select consistent model parameters. In general, used parameters from Umberger et al. (2003) resulted in good prediction of the energy consumption for the performed repetitive submaximal exercise. However, whether this approach (including the chosen parameters) is also applicable for other submaximal contraction levels, other muscles, and other subjects has to be confirmed in future studies.

### 5.2. Conclusion

In conclusion, the core novelty of our approach is that it allows to validate a model of muscular energy expenditure on a muscle specific level *in vivo*. We would like to emphasize that this study shows the potential of this approach, but requires intensive future work to validate the model and the approach across subjects, across different muscles, and across different force levels. If valid across all of these factors, the approach offers new possibilities: The muscle model can predict muscular forces and energy expenditure during defined movement regimes. It is even possible to generate forward-dynamic predictions of movements in musculoskeletal simulations which have not been recorded yet. Combining these methods will allow to investigate energy consumption in dynamic movements in much more realistic detail than before (Umberger and Rubenson, 2011). In the future, muscular energy expenditure models may be combined with the energy consumption of the cardio-vascular system to obtain a better model for whole body energetics (Hochstein et al., 2016).

## Data Availability Statement

The dynamic datasets generated for this study and the model, including all necessary parameters, are included in the article and the Supplementary Material. The original MRS and MRI data will be provided upon request to the authors.

## Ethics Statement

The studies involving human participants were reviewed and approved by Ethics committee of the University Hospital of Jena, Germany. The patients/participants provided their written informed consent to participate in this study.

## Author Contributions

DH, SH, AG, SS, TS, and NS: conceptualization. DH, JS, SH, AG, RR, JR, and NS: methodology. DH, JS, and SS: software. JS, SH, AG, TS, RR, JR, and NS: validation. DH, JS, SH, RR, and NS: formal analysis. DH, JS, SH, AG, and NS: investigation. AG, SS, TS, JR, and NS: resources. DH, JS, SH, and NS: data curation. DH, JS, SH, and NS: writing—original draft. DH, JS, SH, AG, SS, TS, RR, JR, and NS: writing—review and editing. DH, JS, SH, AG, and NS: visualization. DH, SH, and NS: supervision. AG and NS: funding acquisition.

## Conflict of Interest

The authors declare that the research was conducted in the absence of any commercial or financial relationships that could be construed as a potential conflict of interest.
